# Genome-Wide Association Study Singles Out *SCD* and *LEPR* as the Two Main Loci Influencing Intramuscular Fat Content and Fatty Acid Composition in Duroc Pigs

**DOI:** 10.1371/journal.pone.0152496

**Published:** 2016-03-29

**Authors:** Roger Ros-Freixedes, Sofia Gol, Ramona N. Pena, Marc Tor, Noelia Ibáñez-Escriche, Jack C. M. Dekkers, Joan Estany

**Affiliations:** 1 Departament de Ciència Animal, Universitat de Lleida–Agrotecnio Center, Lleida, Catalonia, Spain; 2 IRTA, Genètica i Millora Animal, Lleida, Catalonia, Spain; 3 Department of Animal Science, Iowa State University, Ames, Iowa, United States of America; University of Bologna, ITALY

## Abstract

Intramuscular fat (IMF) content and fatty acid composition affect the organoleptic quality and nutritional value of pork. A genome-wide association study was performed on 138 Duroc pigs genotyped with a 60k SNP chip to detect biologically relevant genomic variants influencing fat content and composition. Despite the limited sample size, the genome-wide association study was powerful enough to detect the association between fatty acid composition and a known haplotypic variant in *SCD* (SSC14) and to reveal an association of IMF and fatty acid composition in the *LEPR* region (SSC6). The association of *LEPR* was later validated with an independent set of 853 pigs using a candidate quantitative trait nucleotide. The *SCD* gene is responsible for the biosynthesis of oleic acid (C18:1) from stearic acid. This locus affected the stearic to oleic desaturation index (C18:1/C18:0), C18:1, and saturated (SFA) and monounsaturated (MUFA) fatty acids content. These effects were consistently detected in *gluteus medius*, *longissimus dorsi*, and subcutaneous fat. The association of *LEPR* with fatty acid composition was detected only in muscle and was, at least in part, a consequence of its effect on IMF content, with increased IMF resulting in more SFA, less polyunsaturated fatty acids (PUFA), and greater SFA/PUFA ratio. Marker substitution effects estimated with a subset of 65 animals were used to predict the genomic estimated breeding values of 70 animals born 7 years later. Although predictions with the whole SNP chip information were in relatively high correlation with observed SFA, MUFA, and C18:1/C18:0 (0.48–0.60), IMF content and composition were in general better predicted by using only SNPs at the *SCD* and *LEPR* loci, in which case the correlation between predicted and observed values was in the range of 0.36 to 0.54 for all traits. Results indicate that markers in the *SCD* and *LEPR* genes can be useful to select for optimum fatty acid profiles of pork.

## Introduction

Intramuscular fat (IMF) content and fatty acid (FA) composition affect both organoleptic quality and nutritional value of pork and, thus, there is increasing interest in including these traits in the selection objectives of pigs bred for quality pork markets. Saturated (SFA) and monounsaturated FA (MUFA) are related to better sensory attributes and technological properties [1], while polyunsaturated FA (PUFA) and MUFA are nutritionally more desirable [2]. Oleic acid (C18:1) is the most abundant FA in pork and it can be regarded as a good target for the simultaneous improvement of organoleptic, technological, and nutritional attributes of pork. Both IMF and FA contents display substantial genetic variation, even within purebred lines [3,4].

During the last decades, a lot of efforts have been put into the detection of quantitative trait loci (QTL) affecting IMF content and FA composition using low-density microsatellite linkage maps [5–8]. However, most of these QTL were detected in experimental crosses and, to our knowledge, they have not been used in commercial breeding programs [9]. An exception is the QTL in the *Sus scrofa* chromosome (SSC) 14 affecting SFA and MUFA in purebred Duroc [10,11], which has been matched to a haplotype of three single nucleotide polymorphisms (SNPs) in the promoter region of the stearoyl-CoA desaturase (*SCD*) gene [12].

The onset of high-density SNP genotyping arrays has enabled a more precise scanning of the genome to detect quantitative trait loci (QTL) and nucleotides (QTN) and to make genomic predictions of breeding values. Genome-wide association studies (GWAS) on FA composition have been performed mostly in experimental crosses [13–15], but there are very few reports for commercial pig populations [16]. Moreover, the accuracy of genomic prediction for IMF content and FA composition in swine has not been assessed. The main objective of this study was to detect genomic variants exhibiting a strong influence on fat content and composition traits, particularly of IMF, in a commercial Duroc population used for producing high quality pork. A secondary objective was to assess whether GWAS on limited high-density SNP data is powerful enough to detect the effect of the *SCD* haplotype on SFA and MUFA, which segregates in the studied population. The potential use of genomic prediction for these traits is discussed in light of the results obtained.

## Materials and Methods

### Ethics Statement

The experimental protocol was approved by the Committee on the Ethics of Animal Experiments of the University of Lleida.

### Animals and data

We genotyped 138 purebred Duroc barrows from the commercial line described in [4] using the PorcineSNP60 v2 Genotyping BeadChip (Illumina, CA). Animals were chosen to be as unrelated as possible. The offspring of 54 sires and 126 dams were chosen to be genotyped. Half of the animals (n = 66, from 29 sires and 57 dams) were born in 2002–2003, and the other half (n = 72, from 25 sires and 69 dams) in 2009–2010. There was no detectable subpopulation structure between pigs from both time periods [17]. All animals were raised in 6 batches (3 batches for each period, with 19 to 26 genotyped animals per batch) under commercial conditions and fed *ad libitum* with a pelleted finishing diet from 160 days of age until slaughter. The average composition of the diet was 16.9% crude protein, 6.6% fiber, and 6.7% fat (C16:0: 20.8%, C18:0: 7.1%, C18:1: 35.4%, C18:2: 27.4%). Animals were slaughtered in the same commercial abattoir at 215.6 (7.9 SD) days of age and 127.7 kg (10.9 SD) of body weight. Carcass backfat thickness (BT, n = 131) at 6 cm off the midline between the third and fourth last ribs was measured by an on-line ultrasound automatic scanner (AutoFOM, SFK-Technology, Herlev, Denmark). Immediately after slaughter, a sample of subcutaneous fat (SF, n = 112) at the level of the third and fourth ribs was collected. After chilling for about 24 h at 2°C, samples of the muscles *gluteus medius* (GM, n = 138) and *longissimus dorsi* at the level of the third and fourth ribs (LD, n = 138) were also collected. The IMF content and FA composition of the samples were determined in duplicate by quantitative determination of the individual FA by gas chromatography [18]. The IMF content was calculated as the sum of each individual FA expressed as triglyceride equivalents [19] and expressed as percentage of fresh sample. Individual and total SFA, MUFA, and PUFA contents were expressed as the percentage relative to total FA. The desaturation ratio of oleic to stearic acid (C18:1/C18:0) and the ratio SFA/PUFA were calculated. Means and range of values observed for each trait are detailed in Table 1. The phenotypes of the genotyped animals were representative of the ranges observed in the whole population. DNA was isolated as described in [12] and used for SNP genotyping with the PorcineSNP60 v2 Genotyping BeadChip (Illumina, CA).

**Table 1:** Mean and range of phenotypic values and posterior means of marker-based additive genetic ($\sigma_{\text{a}}^{2}$) and residual ($\sigma_{\text{e}}^{2}$) variances and heritability (h^2^).

Trait^1^		Phenotypes		Posterior mean of variance components		
		Mean	Range	$\sigma_{\text{a}}^{2}$	$\sigma_{\text{e}}^{2}$	h^2^
Backfat thickness, mm		22.98	12.7–30.1	4.19	7.14	0.37
Muscle *gluteus medius*						
	Intramuscular fat, %	5.07	2.2–9.5	1.00	0.90	0.53
	SFA, %	38.62	34.9–45.5	1.48	0.73	0.67
	MUFA, %	48.41	42.0–52.9	1.76	0.70	0.72
	C18:1, %	44.06	38.1–48.7	1.42	0.62	0.70
	PUFA, %	12.97	8.6–17.7	1.50	1.05	0.59
	C18:1/C18:0	3.65	2.4–4.8	0.087	0.044	0.66
	SFA/PUFA	3.05	2.0–4.7	0.095	0.118	0.45
Muscle *longissimus dorsi*						
	Intramuscular fat, %	3.49	1.5–6.8	0.60	0.50	0.54
	SFA, %	39.58	33.5–48.2	1.81	0.93	0.66
	MUFA, %	49.48	44.8–54.8	1.73	1.00	0.63
	C18:1, %	44.86	39.1–50.5	1.46	1.11	0.57
	PUFA, %	10.94	6.9–16.3	1.99	0.85	0.70
	C18:1/C18:0	3.58	2.1–5.2	0.082	0.046	0.64
	SFA/PUFA	3.76	2.2–7.0	0.132	0.297	0.31
Subcutaneous fat						
	SFA, %	37.94	29.7–44.5	1.41	2.06	0.41
	MUFA, %	44.94	39.1–50.9	1.63	1.49	0.52
	C18:1, %	41.89	36.4–47.3	1.48	1.31	0.53
	PUFA, %	17.12	12.1–22.1	1.22	1.48	0.45
	C18:1/C18:0	3.34	2.3–4.9	0.072	0.069	0.51
	SFA/PUFA	2.26	1.4–3.5	0.050	0.050	0.47

^1^SFA, MUFA, PUFA: saturated, monounsaturated, and polyunsaturated fatty acids; C18:1: oleic acid; C18:0: stearic acid.

Given the GWAS results, two candidate QTN from the most associated regions were genotyped posteriorly for validation purposes. These two additional SNPs were genotyped in an independent set of 853 pigs sampled from all years since 2002 until 2013. The first SNP was the polymorphism AY487830:*g*.*2228T>C* in the *SCD* promoter [12], which was genotyped by real time qPCR (7500 Sequence Detection System, LifeTechnologies) with an allelic discrimination assay (Custom TaqMan SNP Genotyping Assays, LifeTechnologies). The second polymorphism, at exon 14 of the *LEPR* gene (NM_001024587:*c*.*1987C>T*) [20], was genotyped by High Resolution Melt analysis (Luminaris Color HRM Master Mix, Thermo Scientific) in a real time thermocycler (CFX-100, Bio-Rad). Primers used for genotyping these SNPs are detailed in S1 Table. The concentration of leptin in blood plasma at 180 days of age after overnight fasting was analyzed in a subset of animals (n = 73) using a porcine leptin ELISA kit (Diagnostic Systems Laboratories, Inc., Webster, TX) [21]. All samples were evaluated in a double assay. The coefficient of variation between replicates was 7%.

### High-density SNP data quality control

The PLINK software [22] was used to filter out SNPs with minor allele frequency below 0.05 and genotyping rate below 0.95, and individuals with more than 10% missing genotypes. Unmapped SNPs based on the current pig genome assembly *Sus scrofa* Build 10.2 were also excluded. The remaining data comprised 135 individuals and 36,432 SNPs.

### Genome-wide association study

Associations of SNP genotypes with the phenotypes were analyzed using the Bayes B approach [23] implemented in the GenSel software [24]. The basic model was

$$y=\mathrm{Xb}+\sum_{j=1}^{k}z_{j}\alpha_{j}\delta_{j}+e,$$

where **y** is the phenotypes vector, **X** is the incidence matrix relating fixed factors to phenotypes, **b** is the vector of fixed effects, **z**_*j*_ is the vector of (coded) genotypes for a SNP at locus *j* (*j* = 1 to *k*, where *k* is the number of SNPs), α_*j*_ is the allele substitution effect of the SNP at locus *j*, δ_*j*_ is a random 0/1 variable that represents the absence or presence (with prior probabilities π and 1−π, respectively) of SNP *j* in the model for a given iteration of the Markov chain Monte Carlo procedure, and **e** is the vector of random residuals, assumed to be normally distributed. Alternate homozygous genotypes were coded as -10 and 10, heterozygotes as 0, and missing genotypes as the average value in the population. Fixed effects included batch as a class variable and age at slaughter as a covariate. Intramuscular FA composition traits were analyzed with and without IMF content as an additional covariate. Due to the limited number of animals in the study, the prior proportion of SNPs considered to have no effect on the trait (δ_*j*_ = 0) was fixed to π = 0.997, so that the model fitted ~110 SNPs per iteration. Variance components used as priors were estimated as in [4] with the full pedigree (111,305 individuals) and all available phenotypic data (106,276 records for BT and 1,355 records for IMF and FA). A total of 750,000 iterations with a burn-in of 250,000 were run for the analyses. The statistical relevance of the association of individual markers with each trait was evaluated calculating the Bayes Factor for each locus *j* (BF_*j*_) [25,26] as

$${\text{BF}}_{j}=\frac{\frac{{\hat{\text{p}}}_{j}}{1-{\hat{\text{p}}}_{j}}}{\frac{\left(1-\pi \right)}{\pi }},$$

where ${\hat{\text{p}}}_{j}$ is the posterior probability of a SNP at locus *j* of being included in the model at a given iteration of the Markov chain Monte Carlo procedure. Evidence of association was considered "substantial" for BF_*j*_ above 3.2, "strong" above 10, and "decisive" above 100 [27]. Linkage disequilibrium in candidate regions was analyzed using Haploview software [28]. The percentage of genetic variance explained by the individual markers was calculated. To take account of the linkage disequilibrium between SNPs, for each individual we predicted collectively the genomic merit of all SNPs within 1-Mb non-overlapping windows (based on Build 10.2 of the swine genome) and used these values to calculate the proportion of total genetic variance explained by the 1-Mb windows, as detailed in [29]. The average number of SNPs per window was 14.2. Regions that accounted for at least 2.5% of the genetic variance of a trait were considered as candidate regions. Combinations of contiguous windows that explained at least 0.5% of genetic variance were also considered to take into account, in turn, SNPs in linkage disequilibrium spanning more than 1 Mb. Candidate genes in these regions were retrieved from Ensembl (EMBL-EBI) and functional gene annotation was based on Enrichr gene analysis tool [30].

In view of the results, genome-wide associations were reanalyzed after adding *SCD g*.*2228T>C* and *LEPR c*.*1987C>T* to the SNPs of the chip. The association of SNP *LEPR c*.*1987C>T* with leptin concentration in plasma and with the studied traits was analyzed in an independent set of 853 pigs using a model with age at slaughter as a covariate and batch and genotype as class variables. This analysis was performed under a Bayesian setting with Rabbit software [31].

### Genomic prediction

We used the animals born in 2002–2003 as training data to estimate the SNP effects and then to predict the genomic estimated breeding values (GEBV) of the animals born in 2009–2010. The effect of each SNP was estimated using the same procedure as for GWAS but using only the training set (n = 65). The GEBV of an individual *i* in the testing dataset was predicted as

$${\text{GEBV}}_{i}=\sum_{j=1}^{k}{\text{z}}_{ij}{\hat{\alpha }}_{j},$$

where z_*ij*_ is the genotype of animal *i* for a SNP at locus *j* (*j* = 1 to *k*, where *k* is the number of SNPs) coded as above, and ${\hat{\alpha }}_{j}$ is the allele substitution effect estimate for the SNP at locus *j* based on the analysis of the training dataset. The correlation between GEBV and the adjusted phenotypic values of the testing dataset was used as a measure of the prediction accuracy. Phenotypes were adjusted for batch and age at slaughter using a fixed model. In view of the GWAS results, five different sets of SNPs were evaluated for their predictive ability: (1) all SNPs in the chip, (2) only *SCD g*.*2228T>C*, (3) only *LEPR c*.*1987C>T*, (4) only these two SNPs together, or (5) all SNPs in the chip but excluding those in the *SCD* and *LEPR* regions detected by GWAS.

## Results and Discussion

### Genome-wide association study

**Fig 1:**
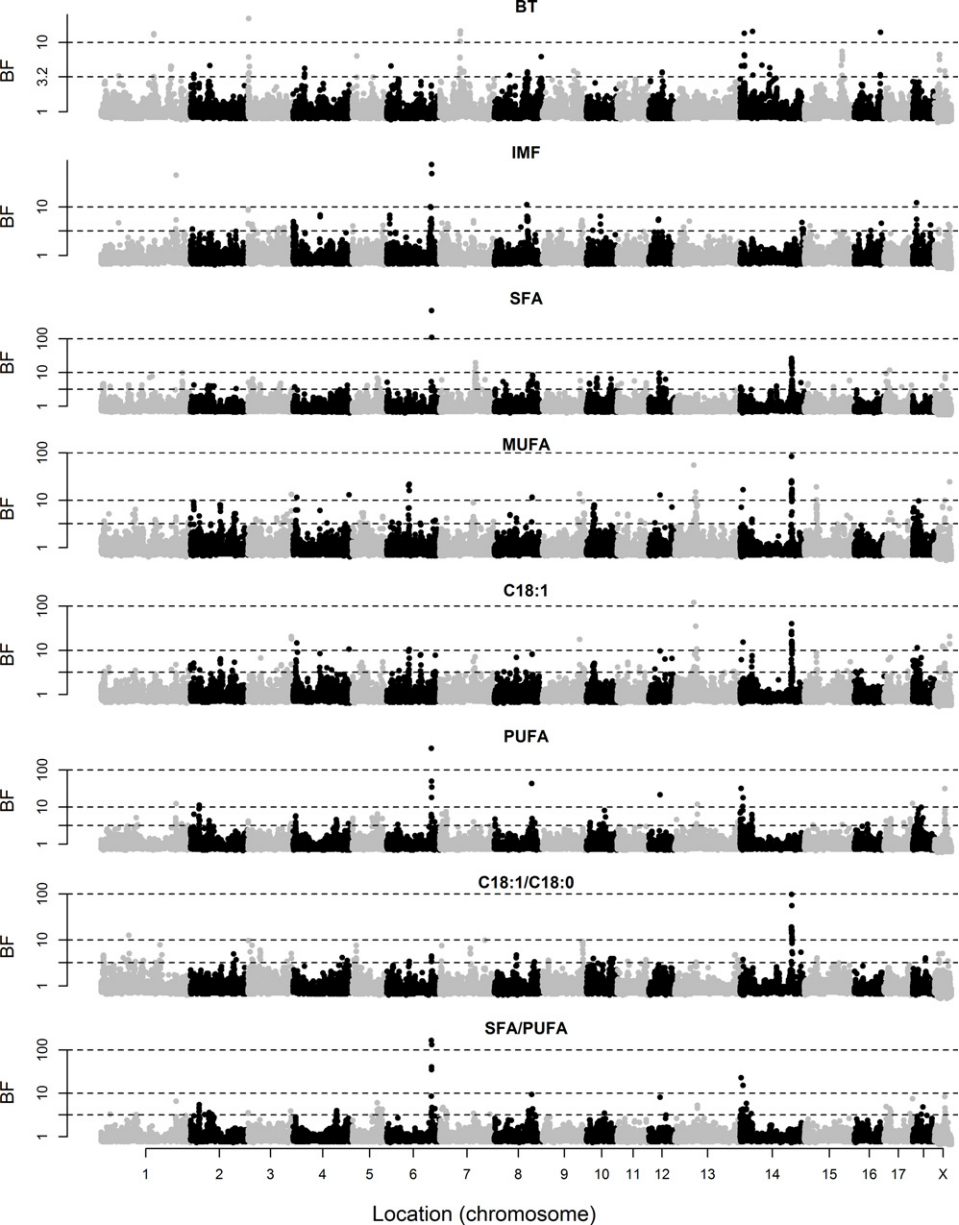
Bayes factors of individual markers for backfat thickness (BT) and intramuscular fat (IMF) content and composition of *gluteus medius*. Fatty acid composition includes saturated (SFA), monounsaturated (MUFA), and polyunsaturated (PUFA) fatty acids, individual oleic acid (C18:1), and the ratios of oleic to stearic acid (C18:1/C18:0) and SFA/PUFA. The discontinuous lines indicate Bayes Factors of 3.2 (substantial evidence), 10 (strong), and 100 (decisive).

**Fig 2:**
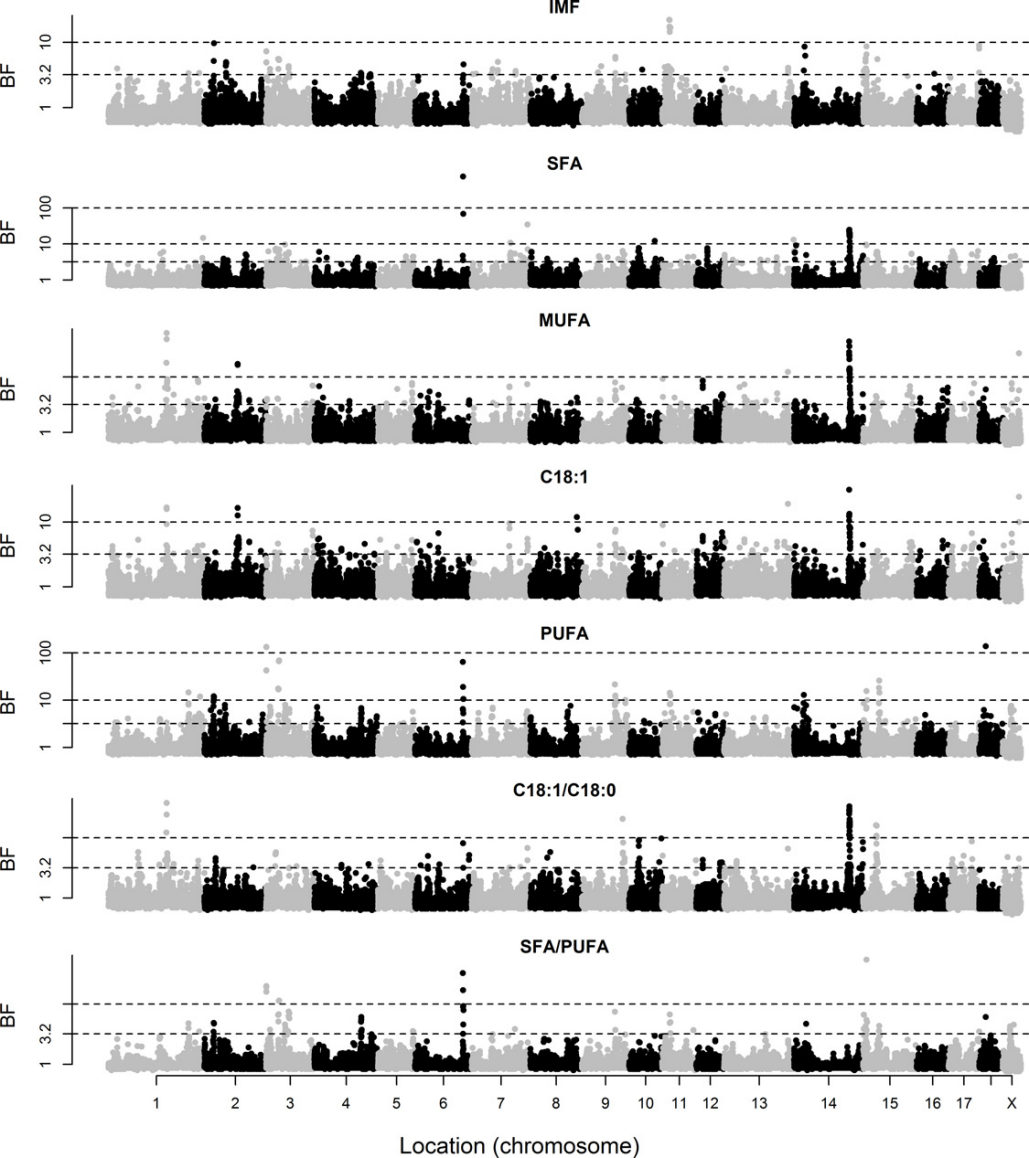
Bayes factors of individual markers for intramuscular fat (IMF) content and composition of *longissimus dorsi*. Fatty acid composition includes saturated (SFA), monounsaturated (MUFA), and polyunsaturated (PUFA) fatty acids, individual oleic acid (C18:1), and the ratios of oleic to stearic acid (C18:1/C18:0) and SFA/PUFA. The discontinuous lines indicate Bayes Factors of 3.2 (substantial evidence), 10 (strong), and 100 (decisive).

**Fig 3:**
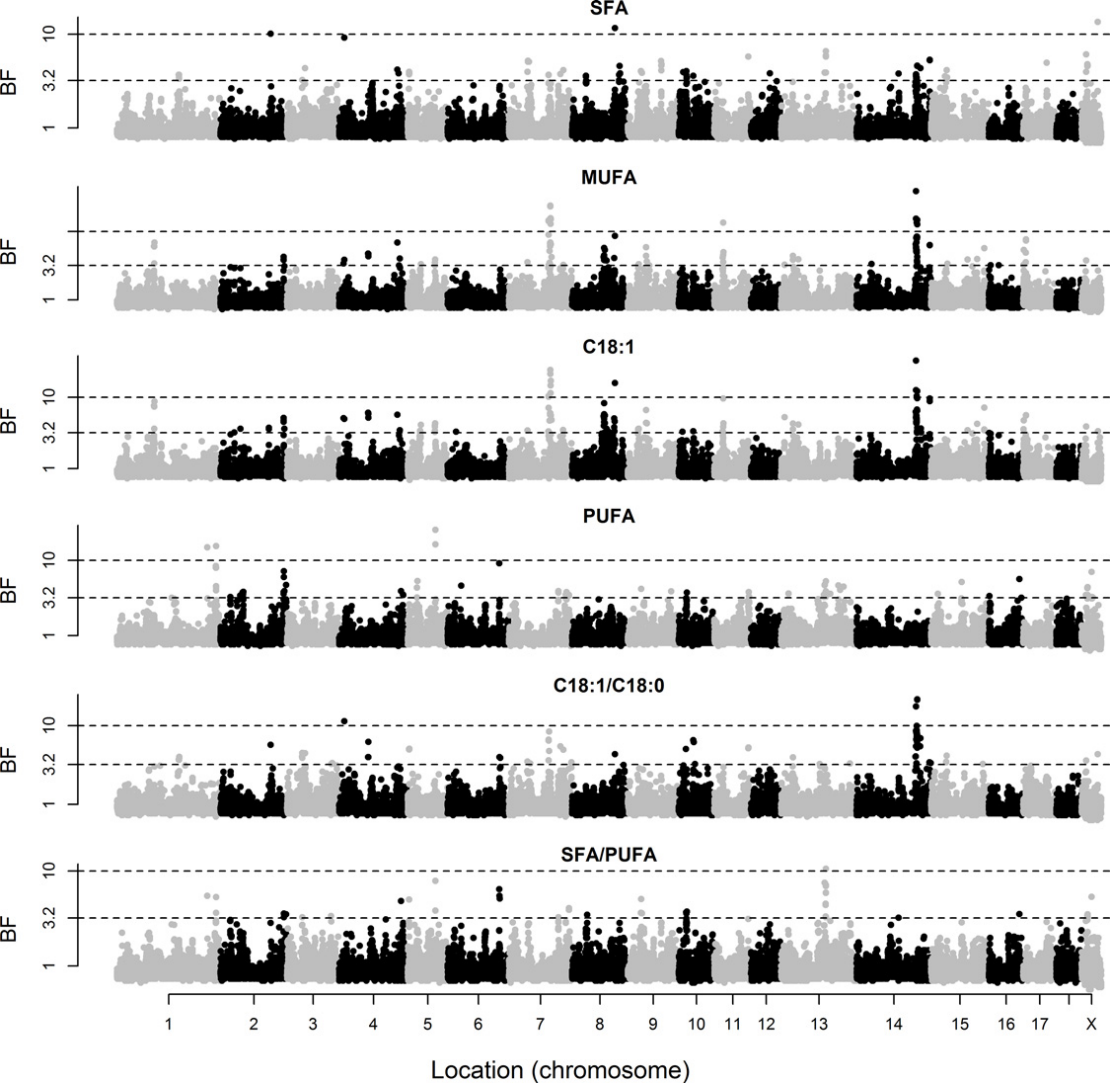
Bayes factors of individual markers for subcutaneous fat composition. Fatty acid composition includes saturated (SFA), monounsaturated (MUFA), and polyunsaturated (PUFA) fatty acids, individual oleic acid (C18:1), and the ratios of oleic to stearic acid (C18:1/C18:0) and SFA/PUFA. The discontinuous lines indicate Bayes Factors of 3.2 (substantial evidence), 10 (strong), and 100 (decisive).

The posterior means of variance components and heritabilities based on the genotypic data are given in Table 1. The marker-based heritabilities ranged from 0.31 to 0.72, indicating that the SNP genotypes explained a relevant proportion of the phenotypic variance of these traits. Marker-based heritabilities were particularly high for FA composition of the two muscles. The BF of individual markers for BT and IMF content and composition of GM are shown in Fig 1, and those of LD and SF are in Figs 2 and 3, respectively. A summary of the regions that explained at least 2.5% of genetic variance is given in Table 2.

**Table 2:** Candidate regions for intramuscular fat content and fatty acid composition traits.

SSC	Position (Mb)	Number of SNPs	Trait^1^	Tissue^2^	Genetic variance^3^ (%)	PPI^4^	Extended region^5^	
							Position (Mb)	Genetic variance^3^ (%)
1	182–183	8	MUFA	LD	3.0	0.31	181–183	3.6
3	1–2	9	PUFA	LD	9.3	0.69	1–3	10.7
			SFA/PUFA	LD	2.5	0.17	1–3	3.2
	29–30	24	PUFA	LD	3.8	0.41	28–30	5.1
5	84–85	25	PUFA AdjIMF	GM	2.5	0.35	-	-
6	135–136	18	IMF	GM	3.1	0.28	135–137	4.9
			SFA	GM	13.2	0.72	135–137	17.6
				LD	12.2	0.72	135–137	14.7
			PUFA	GM	15.4	0.76	135–137	16.9
				LD	2.2	0.31	135–137	2.5
			SFA/PUFA	GM	14.3	0.58	135–137	21.7
				LD	3.0	0.22	135–137	3.6
			SFA AdjIMF	GM	3.0	0.34	135–137	4.1
				LD	6.1	0.47	135–137	7.0
			PUFA AdjIMF	GM	1.0	0.16	135–137	1.2*
				LD	0.4	0.10	135–137	0.4*
			SFA/PUFA AdjIMF	GM	0.9	0.14	135–137	1.2*
				LD	0.5	0.11	135–137	0.9*
7	90–91	15	MUFA	SF	1.7	0.18	89–92	4.1
			C18:1	SF	1.7	0.17	89–92	3.8
9	146–147	20	PUFA AdjIMF	LD	1.6	0.22	146–148	2.5
11	19–20	15	IMF	LD	2.0	0.20	19–21	3.7
12	24–25	15	SFA AdjIMF	GM	2.5	0.39	-	-
13	40–41	15	C18:1	GM	3.3	0.32	-	-
14	121–122	17	SFA	GM	10.1	0.57	120–124	18.2
				LD	9.1	0.54	120–124	17.3
			MUFA	GM	17.5	0.64	120–124	27.8
				LD	12.2	0.53	120–124	24.1
			C18:1	GM	8.4	0.55	120–124	14.7
				LD	2.9	0.31	120–124	8.0
			C18:1/C18:0	GM	31.1	0.71	120–124	45.0
				LD	22.4	0.61	120–124	38.7
			SFA AdjIMF	GM	12.3	0.57	120–124	22.9
				LD	13.6	0.59	120–124	23.7
			MUFA AdjIMF	GM	18.1	0.65	120–124	28.6
				LD	12.8	0.53	120–124	25.2
			C18:1 AdjIMF	GM	4.9	0.35	120–124	16.0
				LD	3.8	0.35	120–124	9.5
			C18:1/C18:0 AdjIMF	GM	30.1	0.70	120–124	44.8
				LD	22.4	0.61	120–124	38.5
	122–123	17	SFA	SF	0.9	0.14	120–124	2.4*
			MUFA	SF	3.7	0.29	120–124	11.5
			C18:1	SF	2.3	0.24	120–124	7.5
			C18:1/C18:0	SF	5.2	0.33	120–124	15.2
15	7–8	17	SFA/PUFA	LD	3.1	0.20	-	-
18	22–23	10	PUFA	LD	3.2	0.37	-	-

^1^IMF: intramuscular fat content; SFA, MUFA, PUFA: saturated, monounsaturated, and polyunsaturated fatty acids; C18:1: oleic acid; C18:0: stearic acid; AdjIMF: trait with intramuscular fat content fitted in the model as a covariate.

^2^GM: muscle *gluteus medius* (n = 135); LD: muscle *longissimus dorsi* (n = 135); SF: subcutaneous fat (n = 112).

^3^Posterior mean of the percentage of total genetic variance explained by the window.

^4^Posterior probability of inclusion (non-zero genetic variance). The average PPI for the windows explaining less than 0.5% of genetic variance was 0.04.

^5^To take account of potential linkage disequilibrium between SNPs, combinations of contiguous 1-Mb windows that explained at least 0.5% of genetic variance were considered. Regions that explained at least 2.5% of genetic variance are shown.

*Below 2.5% but shown due to the importance of the locus.

Strong individual marker associations were detected for carcass BT (BF up to 22.0), but the highest values of explained genetic variance for the 1-Mb windows hardly reached 2% (data not shown). The SSC7 at 46–47 Mb region (1.9% of genetic variance) was the one showing the highest association. No candidate genes were found in this region, but *NFKBIE* (linked with the adipocytokine signaling path) and *SLC29A1* (related to abnormal eating behavior) lie in its vicinity. Interesting candidate genes for BT were found in the SSC8 region at 144–148 Mb (1.3%), containing *AGPAT9*, *GK2*, and *SCD5*, all three related to FA and triglyceride metabolism, and *BMP3*, which is involved in adipogenesis.

**Fig 4:**
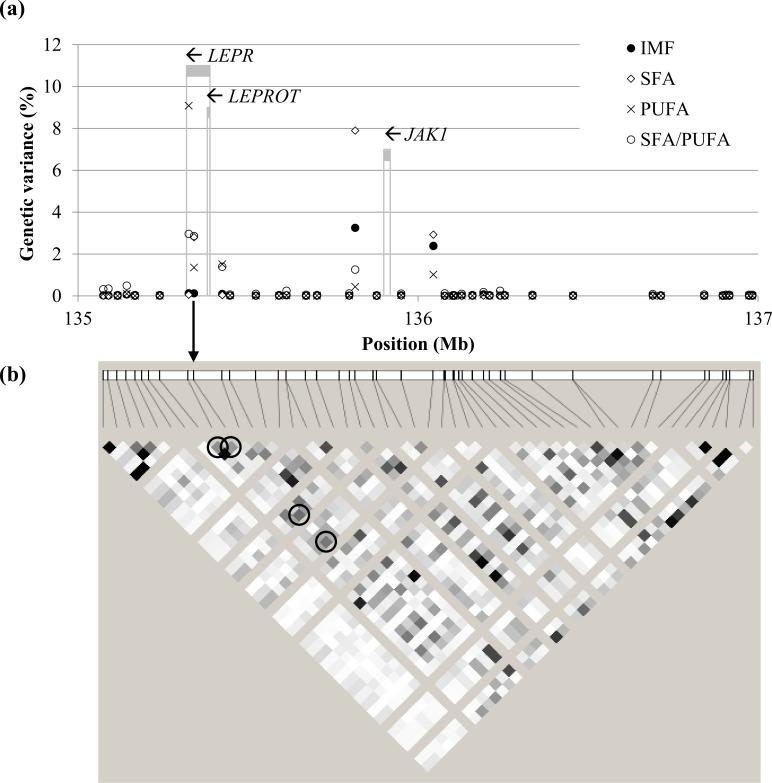
Individual markers in the SSC6 at 135–137 Mb region. Panel (a) shows the percentage of genetic variance explained for intramuscular fat content (IMF), saturated fatty acids (SFA), polyunsaturated fatty acids (PUFA), and SFA/PUFA of muscle *gluteus medius*. The SNP NM_001024587:*c*.*1987C>T* in exon 14 of *LEPR*, indicated with an arrow, is not provided in the chip. Grey stripes indicate the location of candidate genes *LEPR* (tentative), *LEPROT*, and *JAK1*, and arrows indicate sense of transcription. Panel (b) shows the linkage disequilibrium in the region (white: r^2^ = 0; black: r^2^ = 1). The SNP *c*.*1987C>T* is in high linkage disequilibrium with the four SNPs picking up the strongest signals (circled; from left to right: ASGA0089937, ASGA0093565, ALGA0037129, and H3GA0053839).

**Fig 5:**
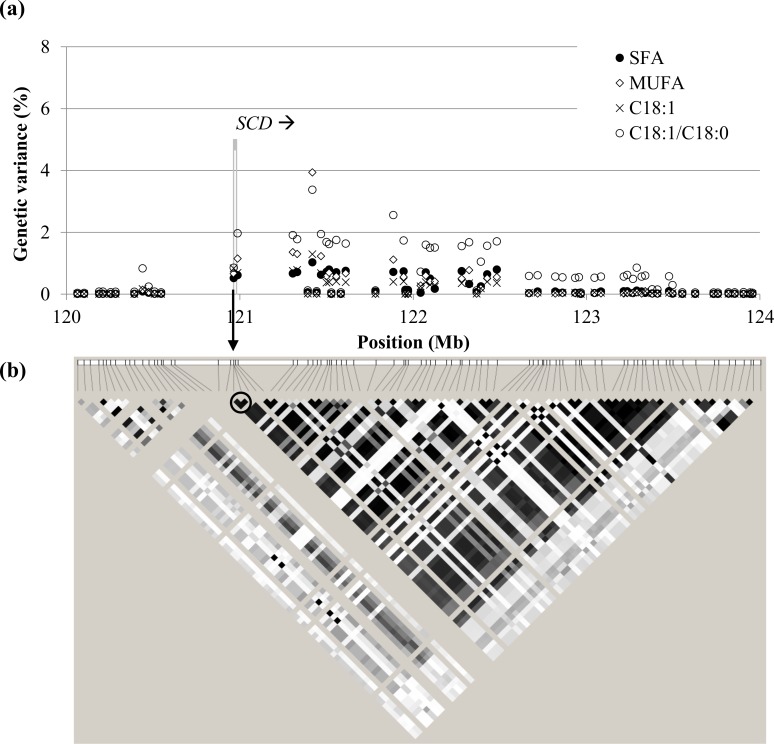
Individual markers in the SSC14 at 120–124 Mb region. Panel (a) shows the percentage of genetic variance explained for saturated fatty acids (SFA), monounsaturated fatty acids (MUFA), oleic acid (C18:1), and the desaturation ratio C18:1/C18:0 of muscle *gluteus medius*. The SNP AY487830:*g*.*2228T>C* from the haplotype described in [12], indicated with an arrow, is not provided in the chip. The grey stripe indicates the location of candidate gene *SCD* and the arrow indicates sense of transcription. Panel (b) shows the linkage disequilibrium in the region (white: r^2^ = 0; black: r^2^ = 1). Circled, the haplotype described in [12], in high linkage disequilibrium with several SNPs downstream associated to the traits.

In contrast, strong signals for IMF content and FA composition traits were located on SSC6 (BF up to 738.3) and SSC14 (BF up to 97.3), which are zoomed in in Figs 4 and 5, respectively. The SNPs with the greatest effect on IMF content in GM were on SSC6 (ALGA0037129 and H3GA0053839 at 135.8 and 136.0 Mb, respectively). These SNPs also had the strongest associations with SFA, followed by several SNPs on SSC14 (121–122 Mb), which, in turn, had the strongest associations with MUFA (as well as markers ALGA0069671 at SSC13 and M1GA0023830 at SSCX). The situation for C18:1 as an individual FA was similar to that of MUFA. The SNP with the greatest effect on PUFA was ASGA0089937 (SSC6 at 135.3 Mb), followed by ASGA0093565 and H3GA0053839 at very close locations (and DRGA0008753 at SSC8). The SNPs on SSC14 at 121–122 Mb and SSC6 at 135–136 Mb also showed the strongest associations with the C18:1/C18:0 and SFA/PUFA ratios, respectively. A similar pattern was observed in LD, except for IMF content, for which the strongest association was found on SSC11 (19.4–20.3 Mb). The signal on SSC14 was also detected for SF composition traits, but not the signal on SSC6.

The window on SSC6 at 135–136 Mb accounted for 3.1% of the genetic variance of IMF in GM, and 4.9% together with the contiguous window at 136–137 Mb. This percentage of genetic variance was explained essentially by four SNPs in linkage disequilibrium (Fig 4). In this region multiple QTL for feed intake, carcass fatness, BT, and IMF have been reported [32]. The 135–136 Mb window includes two overlapping genes, the leptin receptor (*LEPR*) and the leptin receptor overlapping transcript (*LEPROT*), which share the two first exons (Fig 4). Leptin is an adipocytokine that regulates energy intake and expenditure through interaction with its receptor. The *LEPROT* gene encodes a protein that negatively regulates the presence of leptin receptors in the cell surface, decreasing the response to leptin. A non-synonymous polymorphism in the exon 14 of *LEPR* (p.Leu663Phe) has been reported as the probable causative mutation associated with increased feed intake and fatness [20]. The allele T was significantly associated with a lower mRNA expression in the hypothalamus and with the downregulation of the gene *CART* (negative regulation of appetite) and the upregulation of *NPY* (positive regulation of appetite) [33]. The same allele has been also widely and consistently associated with increased fatness traits, including both BT and IMF [34–38]. There is less evidence for the other SNPs found in *LEPR* in pigs [39–41]. The significant signal in this region reaches out about 0.6 Mb, starting in the *LEPR*/*LEPROT* locus and finishing downstream the *JAK1* gene. Interestingly, the signal transductor coded by *JAK1*, which maps to 135.9 Mb (Fig 4), is also involved in the adipocytokine signaling pathway, promoting the leptin-induced transactivation of the satiety neuropeptide *NPY* gene [42]. Mutations in *JAK1* have not been related to fattening traits in pigs before.

In our study, the 135–136 Mb window was also strongly associated with FA composition, both in GM and LD. In particular, the 135–137 Mb extended region explained the greatest percentage of genetic variance for SFA (17.6%), PUFA (16.9%), and SFA/PUFA (21.7%) in GM and, to a lesser extent, in LD (14.7%, 2.2%, and 3.0%, respectively). However, when adjusting for IMF, the genetic variance explained by this region decreased to 0.4–6.1%, indicating that, at least in part, the observed associations of the SNPs in this region with SFA and PUFA are an indirect effect of differences in IMF, as also noted by Galve *et al*. [35]. It is well known that the endogenous synthesis of SFA and MUFA increases with IMF content, which leads PUFA to proportionally decrease [43].

The association of the *LEPR* locus with fat-related traits was further evaluated on an independent set of 853 pigs using the putative causative mutation *LEPR c*.*1987C>T* [20]. Pigs with the TT genotype (allele T frequency = 0.43) for this SNP had higher leptin concentration in plasma, were fatter (both BT and IMF), and had more saturated fat than CC pigs (Table 3). This analysis showed differences between *LEPR* genotypes also for the FA composition of SF, not detected by GWAS but in line with the findings by Muñoz *et al*. [34] and Galve *et al*. [35]. Similar results were obtained with the SNP ASGA0089937 included in the chip as a tag SNP (in intron 3 of the *LEPR* gene; Genbank accession number FN677933.1). In fact, the four SNPs in the chip showing the strongest associations were the ones in greatest linkage disequilibrium with *LEPR c*.*1987C>T* (Fig 4), for which very similar genome-wide associations were found if added to the GWAS. The BF of the association of *LEPR c*.*1987C>T* with IMF was 6.3, and ranged from 38.3 to 93.9 with SFA, PUFA, and SFA/PUFA. Taken together, these results support that a mutation in (or near) the *LEPR* gene affects the leptin regulatory system, similarly to what has been observed in humans [44,45]. As a consequence, it affects feed intake and overall carcass fatness.

**Table 3:** Mean of the estimated marginal posterior distribution of differences between NM_001024587:*c*.*1987C>T* genotypes and probability of the difference being greater than zero (*P*(>0)) for leptin concentration in plasma and fat-related traits in the independent set.

Trait^1^		No. of animals	TT−CC		TT−CT		CT−CC	
			Mean	*P*(>0)	Mean	*P*(>0)	Mean	*P*(>0)
Leptin in plasma, ng/ml		73	+35.50	>0.99	+26.36	>0.99	+9.14	0.89
Backfat thickness, mm		818	+0.94	>0.99	+0.89	>0.99	+0.05	0.57
Muscle *gluteus medius*								
	Intramuscular fat, %	833	+0.77	>0.99	+0.75	>0.99	+0.02	0.55
	SFA, %	839	+1.23	>0.99	+1.10	>0.99	+0.13	0.79
	MUFA, %	839	+0.07	0.61	−0.02	0.45	+0.09	0.70
	PUFA, %	839	−1.28	<0.01	−1.06	<0.01	−0.22	0.09
Muscle *longissimus dorsi*								
	Intramuscular fat, %	267	+0.65	>0.99	+0.76	>0.99	−0.11	0.25
	SFA, %	267	+1.62	>0.99	+1.61	>0.99	0.00	0.51
	MUFA, %	267	−0.15	0.33	−0.12	0.36	−0.03	0.47
	PUFA, %	267	−1.45	<0.01	−1.49	<0.01	+0.04	0.55
Subcutaneous fat								
	SFA, %	272	+1.13	0.98	+0.86	0.95	+0.26	0.75
	MUFA, %	272	−0.09	0.43	−0.08	0.44	−0.01	0.50
	PUFA, %	272	−0.24	0.26	−0.73	0.03	+0.49	0.96

^1^SFA, MUFA, PUFA: saturated, monounsaturated, and polyunsaturated fatty acids.

On the other hand, the region on SSC14 at 120–124 Mb was found to be strongly associated with SFA, MUFA, C18:1, and the desaturation index C18:1/C18:0. The most associated window was located either at 121–122 Mb, for the muscles, or at 122–123 Mb, for SF. This region, which was estimated to capture up to 44.8% of the genetic variance of C18:1/C18:0, corresponds to the location of the *SCD* gene (Fig 5), thereby confirming the association between an haplotype in the promoter of the *SCD* gene and the desaturation of C18:0 to C18:1 that was already found in the same population by Estany *et al*. [12]. The SCD enzyme is rate-limiting for the biosynthesis of MUFA C18:1 from SFA C18:0. Due to the high linkage disequilibrium downstream the *SCD* position (Fig 5), the signal detected spanned 4 Mb and included other genes involved in lipid metabolism, such as *ELOVL3* (responsible for the elongation of long-chain SFA and MUFA), *CHUK* (involved in the adipocytokine signalling pathway), *CYP17A1* (direct role in steroidogenesis), and *PITX3* (related to feeding behavior and to abnormal adipose tissue). The percentages of genetic variance explained by this region for SFA (16.1% in GM and 16.3% in LD), MUFA (27.4% and 22.7%, respectively), C18:1 (14.1% and 8.0%, respectively), and C18:1/C18:0 (41.5% and 37.2%, respectively) were close to those obtained when only accounting for the effect of the *SCD* haplotypes [12]. Moreover, the explained genetic variance did not depend on IMF, which confirms that sequence variation at this locus affects FA composition but not total IMF content [12]. The same association was found, although to a lesser extent, in SF. The BF for *SCD g*.*2228T>C* if included in the analysis was 15.0 for SFA, 15.3 for MUFA, 27.1 for C18:1, and 7.1 for C18:1/C18:0. This polymorphism could affect the expression of the *SCD* gene by disrupting the RXR:RARα and PPARG transcription factor binding sites [12]. However, due to the linkage disequilibrium structure in this region, it was not possible to discriminate the candidate causative SNP from other markers downstream.

Other regions explaining more than 2.5% of genetic variance were found at SSC1, 3, 5, 7, 9, 11, 12, 13, 15, and 18. The region in SSC11 at 19–21 Mb, which was the most associated with IMF in LD (3.7% of genetic variance), contained the genes *RB1*, involved in adipogenesis, and *CYSLTR2*, involved in lipid homeostasis. No functional candidate genes mapped to the other regions.

A previous GWAS performed by Yang *et al*. [15], using a Duroc × Erhualian F_2_ cross and a larger population size, did not reach much different results from ours, with the *SCD* locus being the only reported QTL for major FA in IMF. Zhang *et al*. [16] found an association between the *SCD* locus and FA in LD in commercial Duroc × (Landrace × Yorkshire) hybrids but not in the Chinese breeds Erhualian and Laiwu. Similarly, in the QTL genome scan with microsatellites performed by Uemoto *et al*. [11] in purebred Duroc, the only significant QTL in LD and outer SF layer was SSC14 at 90–113 cM for C18:1 and C18:0. None of their other suggestive QTL for IMF composition matched those found in our study. The same SSC14 QTL was significant for melting point in inner and outer SF layers, a trait that is related to the desaturation degree of fat. No coincident regions were found between our study and GWAS experiments using Iberian × Landrace crossbreds [13,14]. In these studies, neither the *SCD* nor the *LEPR* loci were detected. This result can be expected for the *SCD* locus, because there is no evidence that the investigated *SCD* polymorphism segregates in Iberian, Landrace, or any breed other than Duroc and Large White [12,46]. The *LEPR c*.*1987C>T* polymorphism, though, was first described in an Iberian × Landrace intercross [20], but posterior studies have used mostly Duroc, either purebred [36,37] or crossbred with Landrace × Yorkshire [35,38] or Iberian [34].

Our results highlight that, even if only a limited number of animals are available, GWAS can be a successful strategy to detect polymorphic regions with relatively high effects which are segregating at intermediate frequencies. The minor allele frequencies at the *SCD* and *LEPR* loci were 0.42–0.45, and did not change substantially between the two time periods in the study. Thus, the three genotypes at each loci and time period were similarly represented (S2 Table). Our results showed how GWAS techniques have been able to detect a relevant association already described in the population (at the *SCD* locus) while revealing another one (at the *LEPR*/*LEPROT* locus).

### Genomic selection

We used the animals born in 2002–2003 as training data to re-estimate the SNP effects and then to predict the genomic estimated breeding values (GEBV) of the animals born in 2009–2010. The correlation between the GEBV and the adjusted phenotypic values of the testing dataset are given in Table 4. Note that these correlations should be divided by the square root of heritability of the trait to convert them to accuracies of GEBV as predictors of true breeding values. The correlations of the GEBV based on the 36,432 SNPs in the chip were low (0.04–0.10) for IMF, PUFA, and SFA/PUFA, moderate (0.28) for C18:1, and high (0.48–0.60) for SFA, MUFA, and C18:1/C18:0. The correlations for SFA, MUFA, and C18:1/C18:0 only showed a slight decline when predictions were based only on *SCD g*.*2228T>C* and it improved for C18:1. Similarly, using only *LEPR c*.*1987C>T* raised the correlations of the predictions for IMF, PUFA, and SFA/PUFA to 0.46–0.49, although that for SFA was halved as compared to whole genome predictions. The combination of the two SNPs in *SCD* and *LEPR* provided similar or better accuracies than the whole chip for traits IMF, SFA, PUFA, C18:1/C18:0, and SFA/PUFA, with correlations ranging from 0.43 to 0.50. For MUFA and C18:1, including the *LEPR* genotype as a predictor worsened their prediction, probably because these traits were not associated with *LEPR*. For these two traits, prediction accuracies using only *SCD g*.*2228T>C* were similar to those with the whole chip. Consistently, the rest of SNPs in the chip predicted the phenotypes very poorly.

**Table 4:** Correlations between genomic estimated breeding values and adjusted phenotypes of the 2009-born pigs using the 2002-born as training set and using different sets of SNPs for both training and prediction.

	SNPs used for training and prediction^2^				
Trait^1^	36k	*SCD*	*LEPR*	*SCD*+*LEPR*	36k−*SCD*−*LEPR*
IMF	0.04	-^3^	0.46	0.43	0.03
SFA	0.48	0.38	0.27	0.48	0.17
MUFA	0.50	0.43	-	0.30	0.14
C18:1	0.28	0.36	-	0.15	0.14
PUFA	0.07	-	0.49	0.48	0.04
C18:1/C18:0	0.60	0.54	-	0.50	0.04
SFA/PUFA	0.10	-	0.47	0.46	0.03

^1^IMF: intramuscular fat content; SFA, MUFA, PUFA: saturated, monounsaturated, and polyunsaturated fatty acids; C18:1: oleic acid; C18:0: stearic acid. Determined on muscle *gluteus medius*.

^2^36k: using the 36,432 SNPs in the chip; *SCD*: using only *g*.*2228T>C* from the *SCD* promoter; *LEPR*: using only *c*.*1987C>T* from exon 14 of the *LEPR* gene; *SCD*+*LEPR*: using the two SNPs at the *SCD* and *LEPR* loci; 36k−*SCD*−*LEPR*: all SNPs except the SSC14 at 120–124 Mb (*SCD*) and SSC6 at 135–137 Mb (*LEPR*) extended regions.

^3^A hyphen indicates lack of convergence of the model.

These results, on one hand, confirmed the predictive ability of the SNPs at the *SCD* and *LEPR* loci and, because pigs in the predicted set were separated by a span of seven years from those in the training set, that their effects are consistent across generations. On the other hand, these results suggest that using many SNPs does not necessarily lead to improved predictive ability. To our knowledge, the only attempts to assess the value of genomic prediction for IMF FA composition have been in beef cattle using the BovineSNP50 BeadChip [47,48]. Interestingly, reported correlations in Angus cattle [47] between GEBV and phenotypes using the whole genome SNPs were in line with ours, i.e., very low for PUFA and SFA/PUFA (0.07 and 0.10) and moderate for C18:1, MUFA and SFA (0.26–0.34). In another population of purebred and crossbred Angus cattle [48], *SCD* was also found by GWAS as one of the most influencing genes on IMF FA composition. In this population the correlations between GEBV and phenotypes were also greater for C18:1, MUFA and SFA (0.21–0.28) than for PUFA and SFA/PUFA (0.18–21).

## Conclusions

We have been able to confirm the association of known SNPs at the *SCD* gene with FA composition and to reveal an association that had not yet been detected in the Duroc population under study between the *LEPR*/*LEPROT* region and IMF and FA composition. The SNPs in these two loci can be used conjointly for marker-assisted selection for IMF and FA composition. The other minor candidate regions detected require further research, but they provide a good basis for exploring the development of custom low-density SNP arrays aimed at improving meat quality.

## Supporting Information

S1 Table Primers used for genotyping the single nucleotide polymorphisms (SNP) in the porcine *SCD* gene promoter (AY487830:*g*.*2228T>C*) and exon 14 of *LEPR* (NM_001024587:*c*.*1987C>T*). (PDF)

S2 Table Minor allele frequency and number of animals per genotype within time period for the porcine *SCD* gene promoter (AY487830:*g*.*2228T>C*) and exon 14 of *LEPR* (NM_001024587:*c*.*1987C>T*) SNPs. (PDF)
