# Supplementary material for: Genome-Wide Association Study Singles Out SCD and LEPR as the Two Main Loci Influencing Intramuscular Fat Content and Fatty Acid Composition in Duroc Pigs
Source: PLoS One. 2016 Mar 29;11(3):e0152496. doi: 10.1371/journal.pone.0152496 (PMC4811567; doi:10.1371/journal.pone.0152496)
Supplement: S1 Table — (PDF) [file pone.0152496.s001.pdf]

S1 Table. Primers used for genotyping the single nucleotide polymorphisms (SNP) in the porcine *SCD* gene promoter (AY487830:*g.2228T>C*) and exon 14 of *LEPR* (NM_001024587:*c.1987C>T*).

SNP	Primer name	Sequence 5' → 3'	Final concentration
AY487830:*g.2228T>C*	Primer Forward	CCCTTCTTGGCAGCGAATAAAA	900 nM
	Primer Reverse	CAGGCTGGGTATTTAAAGGCTAGAG	900 nM
	Probe for C allele	VIC-CGACCGTGTCCTGTATT-NFQ	200 nM
	Probe for T allele	FAM-CGACCGTATCCTGTATT-NFQ	200 nM
NM_001024587:*c.1987C>T*	Primer Forward	CAGAGGACCTGAATTTTGGAG	400 nM
	Primer Reverse	CATAAAAATCAGAAATACCTTCCAG	400 nM
