# Supplementary material for: Genome-Wide Association Study Singles Out SCD and LEPR as the Two Main Loci Influencing Intramuscular Fat Content and Fatty Acid Composition in Duroc Pigs
Source: PLoS One. 2016 Mar 29;11(3):e0152496. doi: 10.1371/journal.pone.0152496 (PMC4811567; doi:10.1371/journal.pone.0152496)
Supplement: S2 Table — (PDF) [file pone.0152496.s002.pdf]

S2 Table. Minor allele frequency and number of animals per genotype within time period for the porcine *SCD* gene promoter (AY487830:*g.2228T>C*) and exon 14 of *LEPR* (NM_001024587:*c.1987C>T*) SNPs.

SNP	Time period	f(T)	No. of animals		
			TT	CT	CT
AY487830:*g.2228T>C*	2002-2003	0.42	12	31	23
	2009-2010	0.45	18	29	25
NM_001024587:*c.1987C>T*	2002-2003	0.49	14	34	15
	2009-2010	0.37	10	33	29
